# Efficacy of Platelet-Rich Plasma as an Adjunct to Hair Transplantation: A Systematic Review

**DOI:** 10.7759/cureus.94116

**Published:** 2025-10-08

**Authors:** Sasipaka Sindhusen, Weeratian Tawanwongsri, Chime Eden

**Affiliations:** 1 Division of Dermatology, Bhumibol Adulyadej Hospital, Bangkok, THA; 2 Division of Dermatology, Department of Internal Medicine, School of Medicine, Walailak University, Nakhon Si Thammarat, THA; 3 Division of Dermatology, Jigme Dorji Wangchuck National Referral Hospital, Thimphu, BTN

**Keywords:** androgenetic alopecia, hair transplantation, platelet-rich plasma, scalp, treatment outcome

## Abstract

Hair transplantation is a well-established treatment for androgenetic alopecia (AGA), yet the long-term success of grafts can vary due to multiple influencing factors. Platelet-rich plasma (PRP) has gained attention as a potential adjunctive therapy aimed at enhancing graft survival and promoting hair regrowth. This systematic review evaluated the clinical efficacy of PRP when used alongside hair transplantation. A comprehensive search of Scopus, MEDLINE (via PubMed), and DOAJ was conducted from database inception to June 2025. Eligible studies were prospective clinical trials with control groups that investigated the use of PRP in patients undergoing hair transplantation. Three studies, including two randomized controlled trials and one non-randomized controlled study, met the inclusion criteria, encompassing a total of 217 participants. Across all studies, the addition of PRP was associated with improved outcomes, including increased hair density, enhanced follicle survival, and earlier initiation of hair growth. However, notable heterogeneity was observed in PRP preparation methods, treatment protocols, and outcome measures. Furthermore, none of the studies employed standardized evaluation tools or trichoscopic analysis, limiting the consistency and comparability of findings. While the current evidence suggests that PRP may offer clinical benefits as an adjunct to hair transplantation, further high-quality randomized trials with standardized protocols and long-term follow-up are needed to establish its therapeutic role with greater confidence.

## Introduction and background

Hair transplantation is widely recognized as an effective method for redistributing existing hair in individuals with androgenetic alopecia (AGA) [1]. The procedure involves transferring hair follicles from a donor site to areas with low or absent hair density, thereby enhancing scalp coverage without increasing the total amount of hair. Two common techniques widely recognized and utilized in hair transplantation are follicular unit transplantation (FUT) and follicular unit extraction (FUE). In the FUE technique, individual follicular units are harvested using 0.7-1.1 mm punches, typically from the occipital scalp. When necessary, alternative donor sites such as the beard or chest may be used. The harvested grafts are then implanted into the recipient area to achieve natural-looking hair restoration [2,3]. In the FUT technique, a strip of tissue is excised from the donor area, most commonly the occipital scalp. This strip is then carefully dissected under a microscope to isolate individual follicular units. The prepared grafts are subsequently implanted into the recipient area to restore hair density and coverage [4]. While both FUT and FUE techniques can offer cosmetically acceptable outcomes [5], the long-term success of hair transplantation is influenced by multiple factors, including donor hair characteristics and individual variability in response, particularly the progressive nature of AGA. Since it is a lifelong progressive condition and hair transplantation does not address its underlying pathophysiology, the durability of transplanted grafts may ultimately be limited.

In a study by Kumaresan and Subburathinam [6], 112 male patients who underwent a single session of FUT for Hamilton-Norwood Grade IV AGA were followed up over a four-year period. The results demonstrated varying degrees of hair density retention in the transplanted area: 55.35% of patients experienced a moderate reduction in density, 27.67% showed a slight reduction, 8.03% had a marked reduction, and only 8.92% maintained stable density. These findings suggest that while hair transplantation can yield satisfactory results, the longevity of transplanted grafts may be finite, and there remains a possibility of suboptimal outcomes in some individuals. To ensure durable outcomes, a comprehensive long-term management approach is warranted, typically involving sustained medical therapy and, when necessary, additional surgical interventions. Effective medical treatment supports the preservation of native hair and enhances the overall density achieved through transplantation [7,8].

To achieve satisfactory outcomes in hair transplantation, two key considerations must be addressed. First, graft survival depends on several procedural factors, including proper handling, optimal storage, meticulous surgical technique, and minimal ischemic time [1]. Second, as hair transplantation redistributes rather than regenerates hair, it does not alter the natural progression of AGA, a chronic and progressive condition. Therefore, long-term pharmacological treatment with agents such as minoxidil and finasteride remains essential [9]. In addition, platelet-rich plasma (PRP) injections have gained attention as a promising adjunctive therapy. A systematic review by Donnelly et al. [10] found that six out of nine included studies reported significant improvements in hair density, while five demonstrated increases in hair count. Reported adverse effects were generally mild, with localized pain being the most common, typically transient and manageable.

While PRP therapy as a standalone treatment aims to stimulate existing hair follicles, its application in the post-transplantation setting is intended to support the survival and growth of newly implanted grafts. It may improve the survival rate of transplanted follicles, enhance hair growth, and reduce follicular loss during the catagen phase. Additionally, it may promote faster recovery of the recipient area and activate dormant hair follicles [11,12]. However, the effectiveness of PRP injections specifically in individuals undergoing hair transplantation remains insufficiently established. The studies included in the systematic review by Donnelly et al. [10] evaluated PRP in non-surgical contexts or as a standalone therapy, thereby limiting the generalizability of their findings to post-transplantation applications. Given the increasing interest in incorporating PRP into surgical protocols to enhance graft survival and improve clinical outcomes, further investigation is warranted. The marketing of aesthetic procedures often involves exaggerated claims lacking robust evidence, raising concerns about misinformation. As aesthetic medicine becomes increasingly commodified, promotional strategies tend to prioritize commercial interests over patient welfare. This can result in misleading advertisements that overstate benefits and downplay risks, ultimately distorting patient expectations and decision-making [13,14]. Therefore, the present study aims to conduct a systematic review evaluating the efficacy of PRP as an adjunct to hair transplantation in patients with AGA.

## Review

Methodology

Protocol and Registration

This review was conducted in accordance with the Preferred Reporting Items for Systematic Reviews and Meta-Analyses (PRISMA) guidelines [15]. The protocol was prospectively registered with the International Platform of Registered Systematic Review and Meta-Analysis Protocols (INPLASY) (Registration No. INPLASY202570086). Ethical approval was obtained from the Walailak University Ethics Committee, which granted exemption status (Protocol No. WUEC-25-293-01).

Eligibility Criteria

Eligible studies included clinical experimental prospective studies with a control group, published in English from database inception to June 2025. The population of interest comprised individuals diagnosed with AGA undergoing hair transplantation. The intervention involved the use of PRP as an adjunct to hair transplantation, with the comparison being hair transplantation without PRP. Outcomes of interest included improvement in hair density, hair thickness, graft survival, patient satisfaction, or other measures of clinical efficacy. Studies were excluded if the full text was not accessible, if they were letters to the editor, published in non-peer-reviewed journals, lacked a control group, or were unpublished or classified as grey literature.

Information Sources and Search Strategy

A comprehensive literature search was conducted across three electronic databases: Scopus, MEDLINE (via PubMed), and the Directory of Open Access Journals (DOAJ) to identify relevant studies from database inception to June 2025. The search was limited to articles published in English. A comprehensive literature search was conducted using Scopus, MEDLINE (via PubMed), and DOAJ. An example of the search strategy used in Scopus is as follows:

TITLE-ABS-KEY("platelet-rich plasma" OR PRP) AND TITLE-ABS-KEY("hair transplant*" OR "follicular unit extraction" OR FUE OR "hair restoration") AND ALL("androgenetic alopecia" OR "androgenic alopecia" OR AGA) AND ALL(efficacy OR effectiveness OR outcome* OR "hair density" OR "graft survival" OR "patient satisfaction" OR improvement)

**Figure 1:**
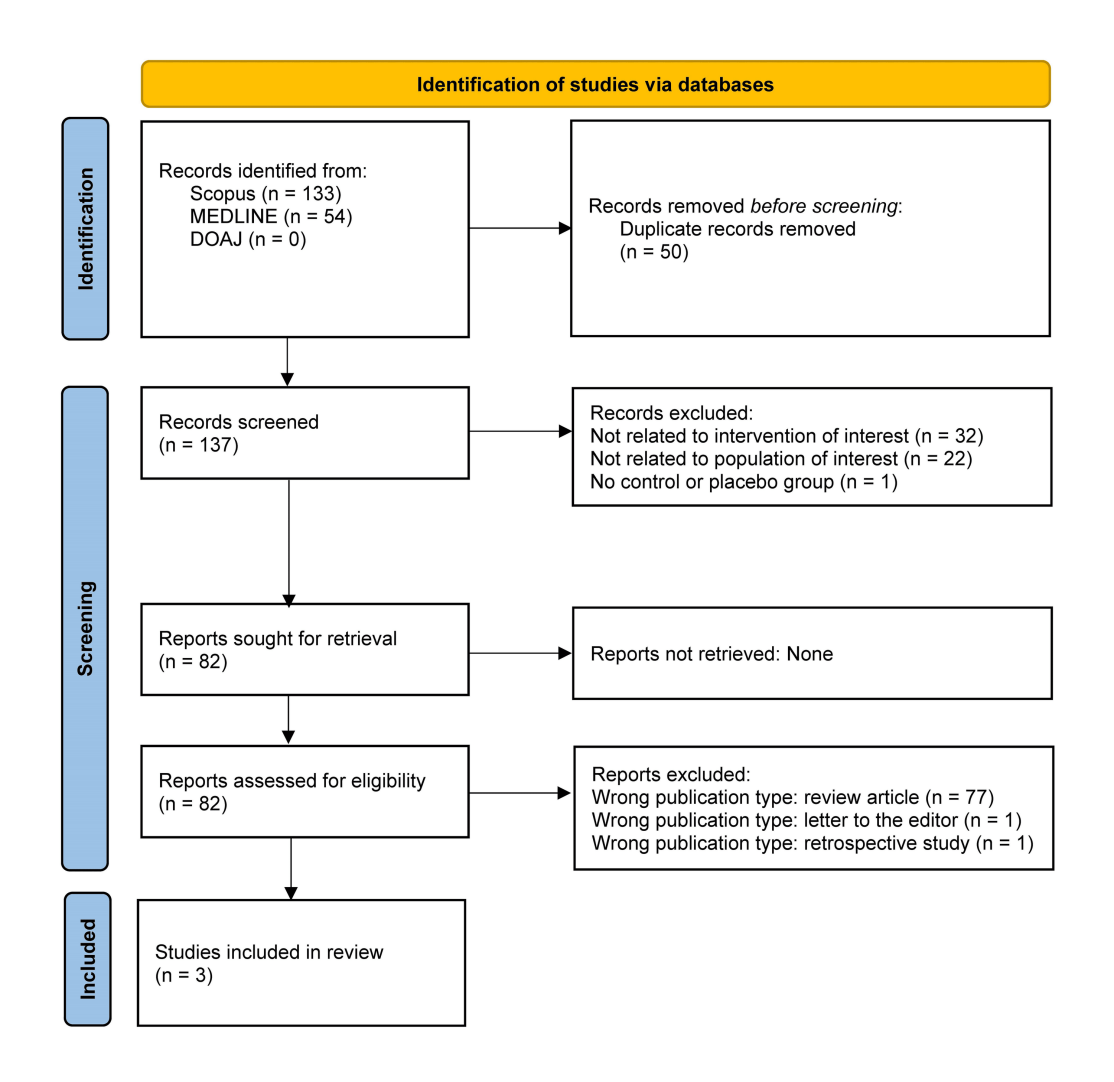
PRISMA flow diagram DOAJ, Directory of Open Access Journals; MEDLINE, Medical Literature Analysis and Retrieval System Online; PRISMA, Preferred Reporting Items for Systematic Reviews and Meta-Analyses

Search strategies for MEDLINE and DOAJ were adapted accordingly using relevant keywords, synonyms, and field-specific syntax appropriate to each database. The complete study selection process is illustrated in Figure 1.

Study Selection and Data Extraction

Two investigators independently screened the titles and abstracts of all retrieved records against the predefined eligibility criteria. This manual process was performed without the use of automation tools or specialized software. Full texts of potentially relevant articles were subsequently reviewed to determine final inclusion. Discrepancies were resolved through discussion until consensus was achieved.

Data extraction was performed independently by both investigators using a standardized data collection form. Extracted variables included: first author and publication year, country of origin, study design, participant characteristics (age, sex, AGA classification), technique of hair transplantation (e.g., FUE parameters, storage method, implantation tools), details of PRP preparation (e.g., blood volume, centrifugation steps, final platelet concentration), type of anesthesia used, reported clinical outcomes (e.g., hair density, follicle survival, regeneration rate, complication rates), and any adverse events. Data were synthesized descriptively due to heterogeneity across study protocols and outcome measures.

Risk of Bias and Methodological Quality Assessment

Two investigators independently assessed the methodological quality and risk of bias of the included studies. Any disagreements were resolved through discussion. For randomized controlled trials (RCTs), the Cochrane Risk of Bias 2 (RoB 2) tool was used, which evaluates five domains and categorizes the overall risk as low, some concerns, or high [16]. For prospective cohort studies, the Risk Of Bias In Non-randomized Studies of Interventions (ROBINS-I) tool was employed, which assesses seven domains and classifies the overall risk as low, moderate, serious, or critical [17]. The risk of bias assessment is summarized in Tables 1, 2.

**Table 1:** Risk of bias assessment for randomized controlled trials using Cochrane Risk of Bias 2 tool (RoB2) D1, Bias arising from the randomization process; D2, Bias due to deviations from intended interventions; D3, Bias due to missing outcome data; D4, Bias in measurement of the outcome; D5, Bias in selection of the reported result.

Author (Year)	D1	D2	D3	D4	D5	Overall
Garg et al. (2016) [18]	Some concerns	Low risk	Low risk	Some concerns	Low risk	Some concerns
Xue et al. (2025) [19]	Low risk	Low risk	Low risk	Some concerns	Low risk	Some concerns

**Table 2:** Risk of bias assessment for non-randomized controlled trials using Risk Of Bias In Non-randomized Studies of Interventions (ROBINS-I) D1, Bias due to confounding; D2, Bias in selection of participants; D3, Bias in classification of interventions; D4, Bias due to deviations from intended interventions; D5, Bias due to missing data; D6, Bias in measurement of outcomes; D7, Bias in selection of the reported result.

Author (Year)	D1	D2	D3	D4	D5	D6	D7	Overall
Zhao et al. (2023) [20]	Serious risk	Moderate risk	Low risk	Moderate risk	Low risk	Moderate risk	Low risk	Serious risk

Software and Statistical Significance

No statistical software was used in this review. All data were synthesized descriptively based on the outcomes reported in the included studies. Statistically significant values (p-values) were extracted directly from the original articles.

Results

This systematic review included three clinical studies comprising a total of 217 patients with AGA, all of which evaluated the efficacy of PRP as an adjunct to hair transplantation. Although the studies differed in methodology, follow-up duration, and outcome measures, all reported favorable outcomes in the PRP groups compared to controls. A summary of study characteristics and findings is presented in Table 3.

**Table 3:** Studies evaluating the efficacy of combining platelet-rich plasma with hair transplantation in patients with androgenetic alopecia AGA, Androgenetic alopecia; FUE, Follicular unit extraction; HT, Hair transplantation; NH, Norwood–Hamilton; PRP, Platelet-rich plasma; RCT, Randomized controlled trial.

Author, year	Country	Study design	Participants	Technique of hair transplantation	PRP preparation and end platelet count, anesthesia used	Efficacy outcomes and adverse reactions
Garg et al., 2016 [18]	India	RCT	40 males, aged 20–55; AGA stage III–VII; follow-up: 6 months	FUE; 40–45 grafts/cm²; implanted within 6 h	20 mL blood; double-spin; ~5–7× platelets; no activator; anesthesia not specified	At 6 months, 100% of the PRP group had >75% follicle growth vs. 20% control (p
Zhao et al. 2023 [20]	China	Non-RCT	147 AGA patients (72 PRP+HT, 75 HT only); follow-up: 8 weeks; baseline characteristics not detailed	Follicles extracted electrically; implantation with gemstone cleft technique	100 mL blood; double-spin; PRP injected 4 weeks post-HT; lidocaine used	Significant improvement in the PRP group vs. control in the hair loss area (0.41 ± 0.21 vs. 1.48 ± 0.38; p = 0.019), pull test (0.21 ± 0.12 vs. 0.59 ± 0.17; p = 0.038), regeneration (3.99 ± 0.56 vs. 6.86 ± 0.84; p = 0.025), and lesion area (5.03 ± 1.01 vs. 8.45 ± 0.97; p = 0.012). No adverse events reported.
Xue et al., 2025 [19]	China	RCT	30 AGA patients (15 PRP, 15 control); mean age ~32; 11M/4F (PRP), 13M/2F (control); NH grade 4; Ludwig grade 1–2; follow-up: 3 months	FUE with 1.0 mm punch; cold graft storage	300–500 mL blood; ~5× platelets; double-spin; PRP injected pre-HT and at 1, 2 months; local anesthesia used	Follicle survival at six months: PRP 82.2% ± 4.1 vs. control 74.0% ± 5.3 (p = 0.002); earlier growth onset (17.7 vs. 20.1 days; p = 0.015); stronger hair (traction test: 13/15 negative in PRP vs. 8/15 in control; p = 0.046). Mild adverse events in both groups; one case of epifolliculitis in the control group.

Collectively, the evidence suggests that PRP consistently enhances follicular outcomes when used alongside hair transplantation. Two RCTs [18,19] and one prospective controlled study [20] reported improvements in hair density, follicle survival, and regeneration when PRP was combined with hair transplantation. Garg et al. [18] demonstrated improved hair density and shaft length, faster resolution of post-procedure redness, and earlier activity in dormant follicles. Similarly, Zhao et al. [20] found that PRP led to significantly better scores in the hair loss area, hair pull test, regeneration index, and lesion area within an eight-week period. Xue et al. [19] observed higher follicle survival rates, earlier onset of hair growth, and stronger hair in the PRP group, with benefits persisting at six months.

A meta-analysis was not conducted due to considerable heterogeneity among the included studies in terms of outcome measures, data formats, and follow-up durations. The studies evaluated different primary endpoints, ranging from categorical assessments of hair density to continuous measures of hair regeneration, lesion area, and follicle survival rates, using varied statistical reporting formats (e.g., means with standard deviations, proportions, or clinical grading scales). Furthermore, follow-up intervals varied from eight weeks to six months, and there were notable differences in PRP preparation techniques, injection protocols, and surgical approaches. Owing to these methodological and clinical discrepancies, statistical pooling was deemed inappropriate.

Discussion

This systematic review highlights promising evidence for the use of PRP as an adjunctive treatment in hair transplantation for patients with AGA. Across the three included clinical studies, two randomized controlled trials and one prospective controlled study involving a total of 217 participants, PRP consistently led to better outcomes compared to standard transplantation alone. Reported benefits included increased hair density, improved follicle survival, faster onset of hair growth, and enhanced regeneration. While the studies varied in design, follow-up periods, and PRP preparation methods, the overall findings support the potential of PRP to enhance the effectiveness of hair transplantation.

A previous meta-analysis examining the effectiveness of PRP in treating androgenic alopecia reported several key findings [21]. First, PRP significantly improved hair density compared to controls, with a mean difference of 25.09 hairs/cm² (95% CI: 9.03-41.15; p = 0.002). However, no statistically significant improvement was observed in hair diameter, with a standardized mean difference of 0.57 (95% CI: -0.23 to 1.38; p = 0.16). Interestingly, subgroup analysis revealed that the beneficial effects of PRP on hair density were more pronounced in male participants, with a statistically significant difference between male-only trials and mixed-sex studies (interaction p-value = 0.02). Several factors have been shown to enhance the response to PRP treatment in AGA. These include a higher number of treatment sessions, higher numbers of platelets, and early-stage alopecia, where patients with mild to moderate hair loss tend to respond better [22, 23]. Younger age and shorter duration of alopecia are also associated with improved outcomes [24]. The therapeutic effects of PRP in AGA are mediated through multiple biological mechanisms [25]. PRP promotes hair regeneration in AGA through multiple mechanisms. Its growth factors, EGF, VEGF, IGF-1, and FGF, stimulate follicular activity, enhance Wnt/β-catenin signaling, and support angiogenesis. EGF aids stem cell renewal and reduces immune-mediated damage, while CCL2 modulates local immunity and recruits growth-promoting M1 macrophages. These actions collectively prolong the anagen phase, reverse follicular miniaturization, and support terminal hair growth.

There is currently no consensus on the optimal PRP preparation protocol for treating AGA, representing a notable gap in the evidence base. While platelet concentrations up to 1.0 × 10⁶/μL are widely regarded as beneficial, some studies have indicated that excessively high concentrations may paradoxically inhibit hair growth [26,27]. It remains unclear whether the single-spin or double-spin centrifugation method is more effective, as no clear agreement has been reached [28]. Despite that, all three studies included in this review employed double-spin protocols; however, their preparation methods varied considerably in key parameters, including the total blood volume processed, which ranged from 20 to 500 mL; the timing of PRP administration, either intraoperative or delayed; and the final platelet concentration, which ranged from approximately five- to sevenfold above baseline levels. None of the studies incorporated activators before injection. These methodological discrepancies likely contributed to variability in clinical outcomes and highlight the need for standardized, well-characterized PRP preparation protocols in future research.

Although all three studies reported positive outcomes following the use of PRP in conjunction with hair transplantation, a key methodological limitation lies in the variability and subjectivity of outcome assessments. None of the studies employed standardized scoring systems, such as the Global Photographic Assessment, Sinclair Scale, or Norwood-Hamilton/Ludwig classifications, to evaluate treatment response in a consistent and reproducible manner [29-31]. In addition, trichoscopic analysis, which offers objective and quantifiable parameters such as hair density, shaft thickness, and follicular unit count, was notably absent [32]. Instead, outcomes were assessed using diverse clinical endpoints, including follicle growth percentages, lesion areas, and hair pull test results, which may be influenced by observer bias and reduce comparability across studies. The lack of standardized and validated assessment tools limits the reliability and generalizability of these findings. Future research should prioritize the use of trichoscopy and uniform scoring systems to enhance the methodological rigor and clinical relevance of treatment evaluations.

All three studies evaluated treatment efficacy over a short to moderate duration, with follow-up periods ranging from eight weeks to six months. While a six-month timeframe is commonly accepted for initial assessment in patients with AGA, it may not fully capture the long-term efficacy of interventions such as PRP. A more comprehensive evaluation would benefit from extending follow-up to at least 12 months, allowing sufficient time for complete hair growth cycles and a more accurate reflection of treatment outcomes [33]. Longer follow-up also facilitates the assessment of sustained clinical improvements, the stability of treatment effects, and the identification of any delayed adverse events. Such extended monitoring is essential for understanding the true therapeutic potential and long-term benefits of PRP in the context of hair restoration.

Several limitations of this systematic review should be acknowledged. First, the review included only three clinical studies, which limits the strength and generalizability of the conclusions. The small sample size also precluded the possibility of conducting a meta-analysis and restricted the ability to perform meaningful subgroup analyses. Second, there was notable heterogeneity among the included studies in terms of PRP preparation protocols. Key variables, such as the volume of blood drawn, centrifugation steps, timing of injection, and final platelet concentration, differed considerably across studies. None of the studies incorporated activators, and variations in surgical techniques and anesthesia use further complicate comparisons. These methodological differences likely contributed to variability in clinical outcomes. Third, the outcome measures used to assess treatment efficacy were inconsistent and largely subjective. None of the studies employed standardized evaluation tools, such as the Global Photographic Assessment, Norwood-Hamilton or Ludwig scales, nor did they utilize trichoscopic analysis, which offers objective quantification of hair density and shaft thickness. The absence of validated and reproducible assessment methods may introduce observer bias and limit the comparability of results. Fourth, the follow-up durations ranged from eight weeks to six months. While these timeframes may capture early treatment effects, they are insufficient to evaluate long-term efficacy and durability. A more comprehensive assessment over 12 months or longer would allow for the full hair growth cycle to be observed and provide a clearer picture of sustained benefits and any delayed adverse effects. Lastly, one of the included studies was a non-RCT, which introduces a higher risk of bias due to potential confounding factors. Although formal risk of bias assessments were conducted, these methodological limitations should be taken into account when interpreting the findings. Future studies should prioritize standardized PRP preparation protocols, employ objective and validated outcome measures, and include longer follow-up periods. Well-designed RCTs are essential to establish the long-term efficacy and clinical value of PRP as an adjunct to hair transplantation.

## Conclusions

This systematic review provides emerging evidence supporting the use of PRP as an adjunct to hair transplantation in individuals with AGA. Across the included studies, the addition of PRP was consistently associated with improved clinical outcomes, including increased hair density, enhanced follicle survival, and earlier initiation of hair growth. These findings underscore the potential of PRP to optimize the results of hair restoration procedures. Nonetheless, the current body of evidence is constrained by several methodological limitations, including small sample sizes, variability in PRP preparation protocols, short follow-up durations, and the absence of standardized outcome measures. These factors limit the generalizability of findings and highlight the need for further research. Future studies should aim to address these gaps through rigorously designed RCTs with well-defined PRP preparation methods, objective and validated assessment tools, and extended follow-up periods. Such efforts are essential to establish the long-term efficacy, safety, and clinical utility of PRP as a standardized adjunctive treatment in hair transplantation.

## References

[1] Jimenez F, Alam M, Vogel JE, Avram M (2021). Hair transplantation: basic overview. J Am Acad Dermatol.

[2] Garg A, Garg S (2021). Overview of follicular extraction. Indian J Plast Surg.

[3] Kerure AS, Deshmukh N, Agrawal S, Patwardhan NG (2021). Follicular unit extraction [FUE] - one procedure, many uses. Indian Dermatol Online J.

[4] Rousso DE, Presti PM (2008). Follicular unit transplantation. Facial Plast Surg.

[5] Gupta AK, Love RP, Harris JA (2020). Old friend or new ally: a comparison of follicular unit transplantation and follicular unit excision METHODS in hair transplantation. Dermatol Surg.

[6] Kumaresan M, Subburathinam DM (2020). Longevity of hair follicles after follicular unit transplant surgery. J Cutan Aesthet Surg.

[7] Kaiser M, Abdin R, Gaumond SI, Issa NT, Jimenez JJ (2023). Treatment of androgenetic alopecia: current guidance and unmet needs. Clin Cosmet Investig Dermatol.

[8] Ly NY, Fruechte S, Hordinsky MK, Sadick N, Arruda S, Farah RS (2023). Medical and procedural treatment of androgenetic alopecia - where are we?. J Am Acad Dermatol.

[9] Panchaprateep R (2024). Medical treatment for androgenetic alopecia. Facial Plast Surg.

[10] Donnelly C, Minty I, Dsouza A (2024). The role of platelet-rich plasma in androgenetic alopecia: a systematic review. J Cosmet Dermatol.

[11] Pathania V, Sood A, Beniwal N, Baveja S, Shankar P, Patrikar S (2023). Randomized control trial to study the efficacy and safety of platelet-rich plasma as intraoperative holding solution in hair restoration surgery: a pilot study. Med J Armed Forces India.

[12] Ranasinghe GC, Khetarpal S (2024). Platelet-rich plasma. Procedures in Cosmetic Dermatology.

[13] Atiyeh BS, Rubeiz MT, Hayek SN (2008). Aesthetic/cosmetic surgery and ethical challenges. Aesthetic Plast Surg.

[14] Lazar CC, Deneuve S (2013). Patients' perceptions of cosmetic surgery at a time of globalization, medical consumerism, and mass media culture: a French experience. Aesthet Surg J.

[15] Sohrabi C, Franchi T, Mathew G (2021). PRISMA 2020 statement: what's new and the importance of reporting guidelines. Int J Surg.

[16] Flemyng E, Moore TH, Boutron I, Higgins JP, Hróbjartsson A, Nejstgaard CH, Dwan K (2023). Using Risk of Bias 2 to assess results from randomised controlled trials: guidance from Cochrane. BMJ Evid Based Med.

[17] Sterne JA, Hernán MA, Reeves BC (2016). Risk of bias in non-randomized studies of interventions (ROBINS-I): detailed guidance. BMJ.

[18] Garg S (2016). Outcome of intra-operative injected platelet-rich plasma therapy during follicular unit extraction hair transplant: a prospective randomised study in forty patients. J Cutan Aesthet Surg.

[19] Xue P, Guo L, Dang E, Dou W, Zeng X, Fan X, Yang Q (2025). A prospective and comparative study to explore the effects of platelet-rich plasma in hair transplantation for patients with androgenetic alopecia. J Cosmet Dermatol.

[20] Zhao L, Cao D (2023). Application of hair transplantation combined with platelet-rich plasma injection for the treatment of androgenic alopecia (Article in French). Eur J Dermatol.

[21] Li M, Qu K, Lei Q, Chen M, Bian D (2024). Effectiveness of platelet-rich plasma in the treatment of androgenic alopecia: a meta-analysis. Aesthetic Plast Surg.

[22] Yuan J, He Y, Wan H, Gao Y (2024). Effectiveness of platelet-rich plasma in treating female hair loss: a systematic review and meta-analysis of randomized controlled trials. Skin Res Technol.

[23] Sasaki GH (2019). Review of human hair follicle biology: dynamics of niches and stem cell regulation for possible therapeutic hair stimulation for plastic surgeons. Aesthetic Plast Surg.

[24] Wu X, Tang Y, Shen N, Wang Z (2025). Analysis on the treatment compliance of patients with androgenetic alopecia and its influencing factors: based on the comparison between microneedle therapy and drug therapy. Arch Dermatol Res.

[25] Abdin R, Zhang Y, Jimenez JJ (2022). Treatment of androgenetic alopecia using PRP to target dysregulated mechanisms and pathways. Front Med (Lausanne).

[26] Li C, Pan L, Yang L, Kong J, Zhang L (2023). An umbrella review of the use of platelet-rich plasma in the treatment of androgenetic alopecia. J Cosmet Dermatol.

[27] Ntshingila S, Oputu O, Arowolo AT, Khumalo NP (2023). Androgenetic alopecia: an update. JAAD Int.

[28] Evans AG, Mwangi JM, Pope RW (2022). Platelet-rich plasma as a therapy for androgenic alopecia: a systematic review and meta-analysis. J Dermatolog Treat.

[29] Guarrera M, Cardo P, Arrigo P, Rebora A (2009). Reliability of hamilton-norwood classification. Int J Trichology.

[30] Sabry HH, Elfalah AA, Mohamed OS, Ibrahim SE (2024). Androgеnеtic alopеcia: a comprеhеnsivе rеviеw of pathogеnеsis, diagnosis, and trеatmеnt options. Benha J Appl Sci.

[31] Chamberlain AJ, Dawber RP (2003). Methods of evaluating hair growth. Australas J Dermatol.

[32] Rossi A, Ferranti M, Magri F (2022). Clinical and trichoscopic graded live visual scale for androgenetic alopecia. Dermatol Pract Concept.

[33] Nestor MS, Ablon G, Gade A, Han H, Fischer DL (2021). Treatment options for androgenetic alopecia: efficacy, side effects, compliance, financial considerations, and ethics. J Cosmet Dermatol.

